# Eliminating Factor H-Binding Activity of *Borrelia burgdorferi* CspZ Combined with Virus-Like Particle Conjugation Enhances Its Efficacy as a Lyme Disease Vaccine

**DOI:** 10.3389/fimmu.2018.00181

**Published:** 2018-02-08

**Authors:** Ashley L. Marcinkiewicz, Ilva Lieknina, Svetlana Kotelovica, Xiuli Yang, Peter Kraiczy, Utpal Pal, Yi-Pin Lin, Kaspars Tars

**Affiliations:** ^1^Division of Infectious Disease, Wadsworth Center, New York State Department of Health, Albany, NY, United States; ^2^Latvian Biomedical Research and Study Centre, Riga, Latvia; ^3^Department of Veterinary Medicine, Virginia–Maryland Regional College of Veterinary Medicine, University of Maryland, College Park, College Park, MD, United States; ^4^Institute of Medical Microbiology and Infection Control, University Hospital of Frankfurt, Frankfurt am Main, Germany; ^5^Department of Biomedical Science, State University of New York at Albany, Albany, NY, United States; ^6^Faculty of Biology, University of Latvia, Riga, Latvia

**Keywords:** Lyme disease, CspZ, *Borrelia*, vaccine, virus-like particles, factor H

## Abstract

The spirochete *Borrelia burgdorferi* is the causative agent of Lyme disease, the most common tick-borne disease in the US and Europe. No potent human vaccine is currently available. The innate immune complement system is vital to host defense against pathogens, as complement activation on the surface of spirochetes results in bacterial killing. Complement system is inhibited by the complement regulator factor H (FH). To escape killing, *B. burgdorferi* produces an outer surface protein CspZ that binds FH to inhibit complement activation on the cell surface. Immunization with CspZ alone does not protect mice from infection, which we speculate is because FH-binding cloaks potentially protective epitopes. We modified CspZ by conjugating to virus-like particles (VLP-CspZ) and eliminating FH binding (modified VLP-CspZ) to increase immunogenicity. We observed greater bactericidal antibody titers in mice vaccinated with modified VLP-CspZ: A serum dilution of 1:395 (modified VLP-CspZ) vs 1:143 (VLP-CspZ) yielded 50% borreliacidal activity. Immunizing mice with modified VLP-CspZ cleared spirochete infection, as did passive transfer of elicited antibodies. This work developed a novel Lyme disease vaccine candidate by conjugating CspZ to VLP and eliminating FH-binding ability. Such a strategy of conjugating an antigen to a VLP and eliminating binding to the target ligand can serve as a general model for developing vaccines against other bacterial infectious agents.

## Introduction

Lyme disease is the most common vector-borne illness in North America and Europe (1). However, no vaccine is currently available for humans. In North America, Lyme disease is caused by the spirochete *Borrelia burgdorferi* sensu stricto, which is transmitted *via Ixodes* ticks (1). Upon tick feeding, spirochetes migrate from the ticks to the vertebrate hosts and infect the skin at the biting site, often resulting in an inflammatory skin lesion, called erythema migrans (2). If untreated, spirochetes disseminate *via* bloodstream to organs, causing disease manifestations including arthritis, carditis, and neuroborreliosis (1). To disseminate to distal tissues, *B. burgdorferi* needs to evade the complement system, an important host innate immune defense mechanism in the blood of vertebrate animals (3). Activation of the complement system results in the formation of C3 convertases, leading to the release of pro-inflammatory peptides, and pathogen opsonization and lysis (3). To avoid self-damage in the absence of pathogens, vertebrate animals produce complement inhibitors, such as Factor H (FH) and FH-like protein 1 (FHL-1, the spliced form of FH). FH and FHL-1 bind to C3b, a component of C3 convertases, which recruits complement protein factor I to degrade C3b and inhibit the formation of these convertases and inactivates the complement system (3).

*Borrelia burgdorferi* produces at least five distinct *C*omplement *R*egulator *A*cquiring *S*urface *P*roteins including CspZ (CRASP-2). CspZ binds to human and mouse FH/FHL-1 to confer serum resistance in a gain-of-function *B. burgdorferi* by inhibiting complement activation on the spirochete surface (4–6). Whereas a *cspZ* deletion mutant of *B. burgdorferi* colonizes tissues at similar levels as its parental wild-type strain (7), mutant strains with transposon insertions in *cspZ*, when co-infected with a library of other transposon-inserted mutants, display reduced colonization of mouse tissues (8). These findings suggest that CspZ contributes a fitness advantage for spirochetes during infection. *cspZ* expression is detectable when spirochetes are in mammalian hosts and *in vitro* cultivation (7, 9), and inoculating mice with CspZ triggers antibody response against this protein (7, 10, 11). Although not all isolates from Lyme disease *Borrelia* species encode *cspZ*, the isolates from *B. burgdorferi* (North American species of Lyme disease spirochetes) all carry this gene (11). The *cspZ* alleles among these *B. burgdorferi* isolates were grouped into three types and share more than 90% of sequence identity (11). These observations suggest that CspZ may have vaccinogenic potential by inducing antibody-mediated bactericidal activity against *B. burgdorferi*. However, immunization with CspZ does not protect mice from infection (7, 11), raising a possibility that CspZ as a vaccine does not induce antibody titers robust enough to kill *B. burgdorferi*.

One strategy to enhance antibody titers and the ability of antibodies in eliminating pathogens is conjugating antigens to virus-like particles (VLPs) (12–14). Though no commercially available vaccines have yet been generated by VLP conjugation, this strategy has been tested in different animal models and shown to trigger greater levels of immune responses [e.g., Ref. (15–18)]. Another strategy is to mutate the immunogens to make them incapable of binding to their binding partners so the epitopes on the binding sites can be exposed (19). We thus modified CspZ by conjugating it to bacteriophage Qβ-derived VLP, combined with eliminating its FH-binding activity to test whether this modified CspZ could be an effective vaccine of Lyme disease. In this study, we demonstrated that vaccination with this modified CspZ induces antibodies that more efficiently eradicate spirochetes *in vitro* and prevents Lyme-associated arthritis and tissue colonization *in vivo*. This proof-of-concept study illustrates novel strategies to generate a potent CspZ-based Lyme disease vaccine. This technique of combining VLP conjugation and eliminating binding to the target ligand can be applied to generate effective vaccines against other infectious agents.

## Materials and Methods

### Ethics Statement

All mouse experiments were performed in strict accordance with all provisions of the Animal Welfare Act, the Guide for the Care and Use of Laboratory Animals, and the PHS Policy on Humane Care and Use of Laboratory Animals. The protocol (Docket Number 16-451) was approved by the Institutional Animal Care and Use Agency of Wadsworth Center, New York State Department of Health. All efforts were made to minimize animal suffering.

### Mouse and Bacterial Strains

Three-week-old male C3H/HeN and Swiss Webster mice were purchased from Charles River (Wilmington, MA, USA) and Taconic (Hudson, NY, USA), respectively. The C3H/HeN mouse strain was utilized as this strain develops manifestations (e.g., arthritis) during *B. burgdorferi* infection and are thus commonly used to test the efficacy of Lyme disease vaccines (20, 21). The *B. burgdorferi* strain B31-A3 used in this study is a clonal isolate of B31 (22) and was grown at 33°C in BSK II complete medium (23). Cultures were tested with PCR to ensure a full plasmid profile prior to use, as previously described (24). *Escherichia coli* strains DH5α, BL21(DE3), and derivatives were grown at 37°C in Luria-Bertani (BD Bioscience, Franklin Lakes, NJ, USA) broth or agar, supplemented with kanamycin (25 µg/mL), ampicillin (100 µg/mL), or no antibiotics when appropriate.

### Generation of VLP-CspZ Proteins

To produce recombinant glutathione-S-transferase (GST)-tagged CspZ proteins, the plasmid pGEX-6P1 encoding the open reading frames lacking the putative signal sequences of *bbh06* (*cspZ*) from *B. burgdorferi* strains B31 (residue 21–236 of CspZ) or an altered open reading frame encoding CspZ-Y207A/Y211A (residue 21–236 of CspZ with tyrosine-207 and -211 replaced by alanine) generated previously (4, 6) was transformed into *E. coli* strain BL21(DE3). The GST-tagged CspZ or CspZ-Y207A/Y211A were produced and purified by GST affinity chromatography as described previously (4, 6) according to the manufacturer’s instructions (GE Healthcare, Pittsburgh, PA, USA). To produce recombinant CspZ proteins without affinity tags for VLP conjugation and vaccination, an cystein has been added to C-termini of both CspZ and CspZ-Y207A/Y211A for coupling these proteins to VLPs as described (25, 26). The genes encoding these proteins were cloned into the pETm_11 expression vector (EMBL) encoding an N-terminal 6xHis-tag followed by a TEV protease cleavage site, resulting in an amino acid sequence of MHHHHHHENLYFQS-CspZ-GSGC. *E. coli* XL1-Blue cells were transformed with the plasmids encoding *cspZ* or *cspZ-Y207A/Y211A*. The transformations were verified by sequencing the plasmid DNA extracted from isolated colonies. *E. coli* BL21(DE3) cells were transformed with these plasmids and grown in modified 2×TY medium at 37°C until mid-log phase. The cultures were then induced to produce CspZ with 0.2 mM isopropyl thio-β-d-galactoside, and grown overnight at 20°C. The cells were lysed by sonication. After removing the debris, the supernatant was loaded onto a HisTrap FF column (GE Healthcare, Chicago, IL, USA) and eluted with 300 mm imidazole at pH 7.5. The 6×His tag was removed by incubation with TEV protease at 4°C overnight. Imidazole was removed by dialyzing the proteins in PBS buffer. The protease, the digested 6×His tag, and un-cleaved proteins were removed using an additional round of HisTrap FF column purification. The purified protein fraction was concentrated using an Amicon centrifugal filter unit (Millipore, Billerica, MA, USA). The purity of the recombinant proteins was evaluated by SDS-PAGE. The bacteriophage Qβ-derived VLPs were generated as previously described (27). Purified CspZ proteins were chemically conjugated to VLPs with SMPH (Succinimidyl-6-[(β-maleimidopropionamido) hexanoate]) following the manufacturer’s protocol (ThermoFisher, Waltham, MA, USA). The unbounded protein was removed using a Superdex200 size exclusion column (GE Healthcare).

### FH Binding Assays by ELISA

Quantitative ELISA for mouse FH binding by CspZ proteins was performed similarly to that previously described (28). Basically, 1 µg of BSA (negative control) or mouse FH (MyBiosource, San Diego, CA, USA) was coated onto microtiter plate wells. One hundred microliters of increasing concentrations (0.03125, 0.0625, 0.125, 0.25, 0.5, 1, 2 µM) of GST (negative control) or a GST tagged wild-type or mutant CspZ protein, including CspZ or CspZ-Y207A/Y211A were then added to the wells. To detect the binding of GST-tagged proteins, mouse anti-GST tag (Sigma-Aldrich, St. Louis, MO, USA; 1:200) and HRP-conjugated goat anti-mouse IgG (Promega, Madison, WI, USA; 1:1,000×) were used as primary and secondary antibodies. The plates were washed three times with PBST (0.05% Tween 20 in PBS), and 100 µL of tetramethyl benzidine (TMB) solution (ThermoFisher, Waltham, MA, USA) were added to each well and incubated for 5 min. The reaction was stopped by adding 100 µL of 0.5% hydro sulfuric acid to each well. Plates were read at 405 nm using a Tecan Sunrise Microplate reader (Tecan, Morrisville, NC, USA).

### Mouse Immunization

Twenty-five micrograms of VLP, CspZ, VLP-CspZ, or VLP-CspZ-Y207A/Y211A were thoroughly mixed with 50 µL TiterMax Gold adjuvant (Norcross, GA, USA), which was utilized because it has been reported to induce higher and longer lasting titers with fewer injections than the other adjuvants (29). This vaccination was then inoculated into C3H/HeN mice intraperitoneally. Mice inoculated with 100 µL PBS were included as a negative control. Mice received boosters of the same composition at 14 and 28 days post immunization, for a total of three immunizations over 6 weeks (Figure S1A in Supplementary Material).

### Quantification of Anti-CspZ Titers with ELISA

Forty-two days post immunization, 100 µL blood was collected from 10 mice *via* submandibular bleeding to isolate serum. The sera were used to determine the titers of immunoglobulin M or G against CspZ using kinetic ELISA as previously described (30). In brief, microtiter plate wells were coated with 1 µg of recombinant CspZ. After blocking with 5% BSA (Sigma-Aldrich) in phosphate-buffered saline, 50 µL of mouse serum diluted 1:100, 1:300, 1:900, 1:1,800, 1:3,600, 1:7,200, 1:144,000, or 1: 288,000 was added to each well. HRP-conjugated goat anti-mouse IgM or IgG (1:20,000; Bethyl, Montgomery, TX, USA) and 50 µL of tetramethyl benzidine (TMB) solution (ThermoFisher, Waltham, MA, USA) were subsequently added into the wells, and the binding was detected at 620 nm for 10 cycles of 60 s kinetic intervals with 10 s shaking duration in a Sunrise absorbance ELISA plate reader (Tecan, Männedorf, Switzerland). The greatest maximum slope of optical density/minute per sample was multiplied by the respective serum dilution factor to indicate the antibody titers (arbitrary Unit).

### *B. burgdorferi* Bactericidal Activity of Serum from Immunized Mice

Forty-two days post immunization, 100 µL blood was collected from five mice *via* submandibular bleeding to isolate serum. The mouse sera were used to determine the bactericidal activity against *B. burgdorferi* with serum bactericidal assays modified from previous studies (31, 32). Prior to determining the bactericidal activity, these mouse sera were heat treated at 56°C for 30 min to inactivate the complement system in these sera. Then, 50 µL of diluted mouse serum (1:20, 1:40, 1:80, 1:160, 1:320, 1:640, 1:1,280, and 1:2,560) was mixed with 10 µL of complement preserved guinea pig serum (guinea pig complement, Sigma-Aldrich, # S1639) or heat-inactivated guinea pig serum (negative control) as well as *B. burgdorferi* strain B31-A3 (5 × 10^5^ cells/mL) in 40 µL of BSK II complete medium and then incubated at 33°C for 24 h. Surviving spirochetes were quantified by directly counting only motile spirochetes using dark-field microscopy. The survival percentage was the proportion of serum-treated to untreated *B. burgdorferi*. The 50% borreliacidal titer representing the serum dilution rate that effectively killed 50% of spirochetes was calculated using dose–response stimulation fitting in GraphPad Prism 5.04 (GraphPad Software, La Jolla, CA, USA).

### Passive Immunization of Mice

Six naive Swiss Webster mice were intraperitoneally inoculated with 100 µL of pooled serum from VLP-, CspZ-, VLP-CspZ-, or VLP-CspZ-Y207A/Y211A-immunized mice (Figure S1B in Supplementary Material). Six mice inoculated with pre-immune serum were included as negative control. They were then challenged subcutaneously with 10^4^ infectious *B. burgdorferi* strain B31-A3 the next day. Mice were euthanized at 14 days post infection, and the inoculation site of skin, heart, tibiotarsus joints, bladder, and ears were collected and then placed at 33°C in BSK medium supplemented with antimicrobial agents (rifampin at 50 mg/mL, phosphomycin at 200 mg/mL, and amphotericin B at 8 mg/mL). Cultures were checked weekly for 4 weeks using dark-field microscopy to determine whether the live *B. burgdorferi* was present. A mouse was considered infected when at least one culture was positive.

### Active Immunization of Mice and Tibiotarsus Joint Measurement

Forty-two days post immunization, the diameter of both tibiotarsus joints was measured with Digimax calipers (Bel-Art, Wayne. MJ, USA). Mice were then subcutaneously needle-infected with 10^4^ *B. burgdorferi* strain B31-A3 suspended in 100 µL BSK II incomplete medium (Figure S1C in Supplementary Material). Negative control mice were injected with an equal volume of BSK II incomplete medium. The diameter of both tibiotarsus joints were measured prior to infection and then were re-measured 7 and 14 days post infection, and the diameters from each mouse averaged as Lyme-induced joint swelling is detectable as early as these time points (33).

### Histopathology of *B. burgdorferi* Infected Mice

Three infected mice per vaccination type and three uninfected mice were sacrificed 14 days post infection to assess arthritis and carditis. Thus, tibiotarsus joints were collected for tissue histopathology. Tissues were fixed for 48 h in 10% neutral-buffered formalin, and subsequently decalcified for 1 week in 10% formic acid. Fixed tissues were prepared as slides stained with hematoxylin and eosin (Wadsworth Histopathology Core Facility, NYS Department of Health, Albany, NY, USA). Arthritis was evaluated in a blind fashion as described previously (30).

### Quantification of *B. burgdorferi* Burden in Infected Mouse Tissue with Quantitative PCR (qPCR)

To quantify *B. burgdorferi* bacterial burden, 10 mice per vaccination type were sacrificed at 28 days post infection, and inoculation site of the skin, knees, and hearts were collected. DNA was purified from tissues using either DNeasy Blood and Tissue Kit (Qiagen, Valencia, CA, USA) or EZ-10 Spin Column Animal Genomic DNA Mini-Prep Kit (Bio Basic, Inc., Markham, ON, Canada). The quantity and quality of DNA were assessed by measuring the concentration of DNA and the ratio of the UV absorption at 280 nm to 260 nm using a Nanodrop 1000 UV/Vis spectrophotometer (Thermo Fisher Scientific, Waltham, MA, USA). qPCR was then performed to quantitate *B. burgdorferi* burden, as described previously (30). In brief, *B. burgdorferi* genomic equivalents were calculated using an Applied Biosystems 7500 Real-Time PCR system (Thermo Fisher Scientific, Waltham, MA, USA) in conjunction with PowerUp™ SYBR^®^ Green Master Mix (Thermo Fisher Scientific, Waltham, MA, USA), based on amplification of the *B. burgdorferi recA* gene using primers BBRecAfp (5′-GTGGATCTATTGTATTAGATGAGGCTCTCG-3′) and BBRecArp (5′-GCCAAAGTTCTGCAACATTAACACCTAAAG-3′). Cycling parameters were 50°C for 2 min, 95°C for 10 min, and 45 cycles of 95°C for 15 s, and 60°C for 1 min. The number of *recA* copies was calculated by establishing a threshold cycle standard curve of a known number of *recA* gene extracted from B31-A3, and burdens were normalized to 10 ng of total DNA.

### Statistical Analyses

Significant differences between groups were determined with one-tailed Fisher Exact Probability Test or one-way ANOVA and *post hoc* tests (GraphPad Software, La Jolla, CA, USA). A *p*-value < 0.05 was used to determine significance.

## Results

### The Generation and Verification of VLP-Conjugated CspZ Proteins

**Figure 1:**
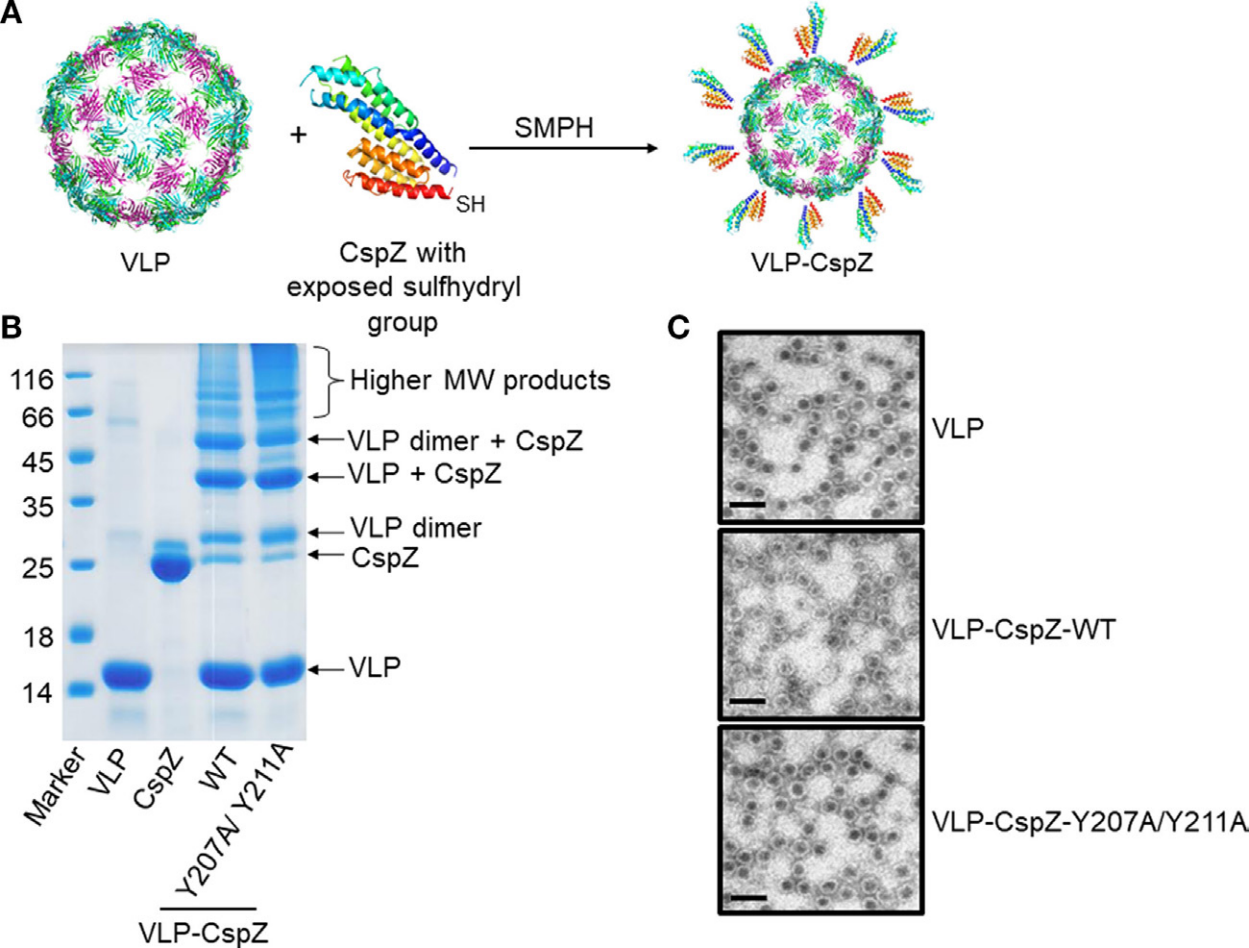
The generation and verification of virus-like particle (VLP)-conjugated CspZ proteins. **(A)** Purified wild-type CspZ or CspZ-Y207A/Y211A with an exposed a sulfhydryl group and virus-like particles (“VLP”) generated from Qβ phage were mixed with Succinimidyl 6-((beta-maleimidopropionamido) hexanoate) (“SMPH”) to crosslink VLP and each of these CspZ proteins (“VLP-CspZ”). **(B)** The sizes and purity of each conjugate were determined by a 15% SDS-PAGE. The molecular marker (kD) is in lane 1, followed by preparations of VLP (lane 2, “VLP”), CspZ (lane 3, “CspZ”), VLP-CspZ (lane 4, “VLP-CspZ-WT”), and VLP-CspZ-Y207A/Y211A (lane 5, “VLP-CspZ-Y207/Y211A”). The arrows identify known protein products as indicated. **(C)** Representative images of VLPs generated in this study. EM analysis of freshly purified VLP (“VLP”), VLP-CspZ (“VLP-CspZ-WT”), or VLP-CspZ-Y207A/Y211A (“VLP-CspZ-Y207A/Y211A”) following negative staining with 1% uranyl acetate. Scale bar = 100 nm.

We re-evaluated the potential of CspZ as a vaccine by utilizing two different strategies: conjugating CspZ with VLP (“VLP-CspZ”) and further modifying CspZ to eliminate its FH-binding activity (“VLP-CspZ-Y207A/Y211A”). The point mutant CspZ-Y207A/Y211A has been shown with no human FH-binding activity (6). Because murine model of Lyme disease infection was used in this study to test the vaccine efficacy of these CspZ-derived proteins, we measured the mouse FH-binding activity of CspZ-Y207A/Y211A by quantitative ELISA. As shown in Figure S2 in Supplementary Material, CspZ binds to mouse FH in a dose dependent manner consistent with a previous finding (5) whereas CspZ-Y207A/Y211A does not bind to mouse FH. In addition, the VLP utilized in this study was derived from the RNA bacteriophage Qβ, which has been used for vaccine development studies in different animal models (15–18). We attached recombinant CspZ proteins to VLPs by adding an engineered C-terminal cysteine to CspZ and linking it to surface-exposed lysine amino groups of VLPs using SMPH (Succinimidyl 6-((beta-maleimidopropionamido)hexanoate)) cross-linker (Figure 1A). The efficiency of coupling was verified by SDS-PAGE. Similar to other VLP-conjugated proteins (25), oligomerized coat protein of VLP was observed on SDS-PAGE (Figure 1B). VLP integrity was maintained as observed under electro-microscopy (Figure 1C).

**Figure 2:**
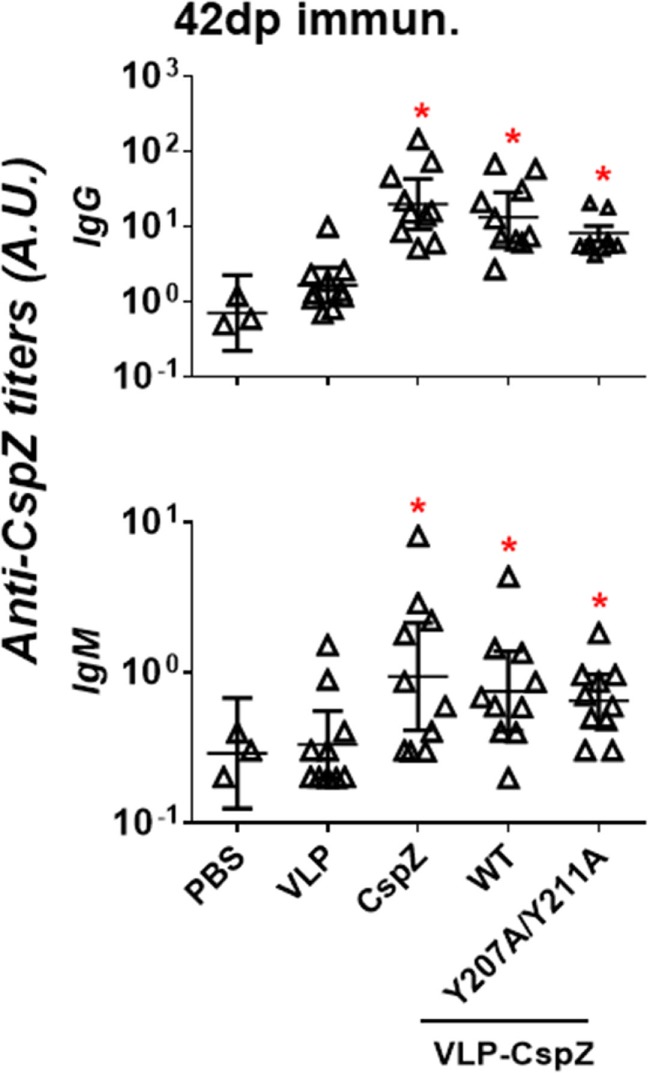
Immunization of CspZ, VLP-CspZ, and VLP-CspZ-Y207A/Y211A triggered undistinguishable antibody response against CspZ. Ten C3H/HeN mice were inoculated with PBS (“PBS”), virus-like particle (“VLP”), CspZ (“CspZ”), VLP-CspZ (“VLP-CspZ-WT”), or VLP-CspZ-Y207A/Y211A (“VLP-CspZ-Y207A/Y211A”), and the serum was obtained at 42 days post inoculation. The serum collected from three C3H/HeN mice at 42 days post inoculation of PBS was also included as negative control. The levels of IgG (*top panel*) and IgM (*bottom panel*) against CspZ were determined using quantitative ELISA as described in Section “Materials and Methods.” Data shown are the mean ± SD of three (PBS) or ten (all others) mice per group. Statistical significances (*p* < 0.05) of differences in antibody titers relative to PBS-inoculated mice were determined using a one-way ANOVA test and are indicated (“*”).

Vaccinating mice with CspZ, VLP-CspZ, or VLP-CspZ-Y207A/Y211A induced similar levels of anti-CspZ antibodies. To examine whether the conjugation of CspZ with VLP and/or the elimination the ability of CspZ to bind FH enhances its immunogenicity, we immunized C3H/HeN mice with PBS, VLP, CspZ, VLP-CspZ, or VLP-CspZ-Y207A/Y211A (Figure S1 in Supplementary Material). This mouse strain, though deficient of TLR signaling (34), has been included in this study as C3H/HeN mice develop apparent manifestations (e.g., arthritis) during *B. burgdorferi* infection (35). Therefore, this mouse model has been commonly utilized for the Lyme disease vaccine study to recapitulate associated manifestations in humans (20, 21). We quantitatively measured the levels of antibodies against CspZ in the sera from these mice using ELISA. As expected, the titers of anti-CspZ IgG and IgM in VLP-treated mice were not different from PBS-treated mice (Figure 2). Consistent with previous findings (7, 11), vaccination with CspZ elicited antibody response against CspZ, which was 5- (for IgM) to 10-fold (for IgG) higher than PBS- or VLP-inoculated mice (Figure 2). VLP-CspZ and VLP-CspZ-Y207A/Y211A vaccinations also induced anti-CspZ antibodies [5- (for IgM) to 10-fold (for IgG) greater than PBS- and VLP-treated mice; Figure 2]. However, the anti-CspZ antibody responses induced by CspZ, VLP-CspZ, and VLP-CspZ-Y207A/Y211A vaccination were not different, suggesting that conjugating CspZ to VLP or eliminating FH-binding activity of this protein does not increase the total antibody response against CspZ.

**Figure 3:**
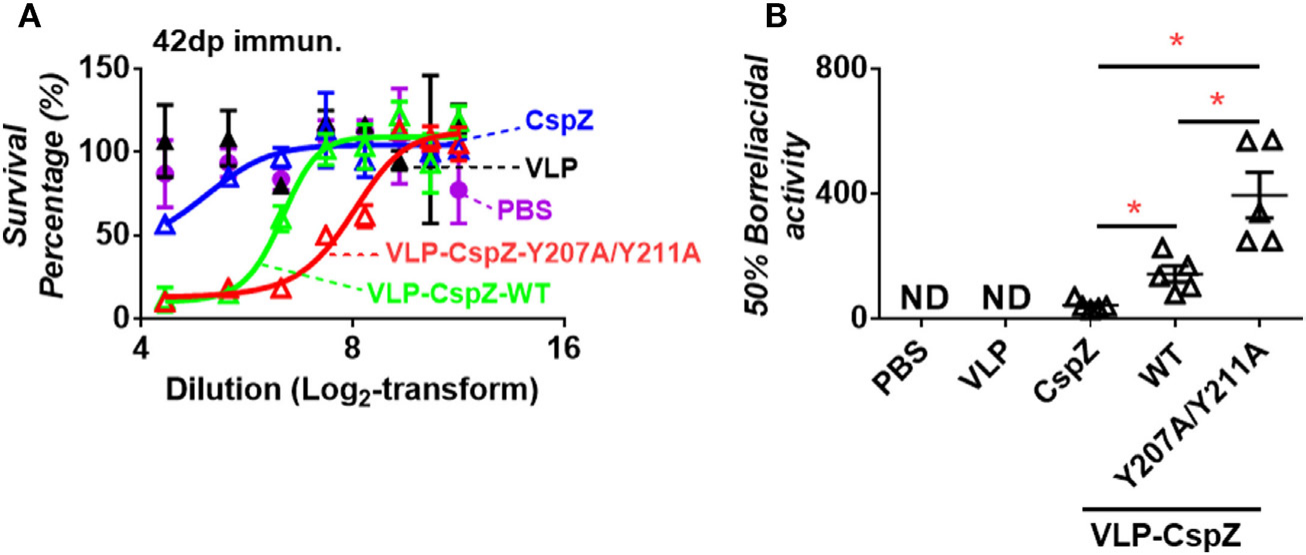
Serum from mice immunized with VLP-CspZ-Y207A/Y211A had more robust levels of bactericidal activity than VLP- or VLP-CspZ-vaccinated mice. Serum collected five C3H/HeN mice at 42 days post inoculation of virus-like particle (“VLP”), CspZ (“CspZ”), VLP-CspZ (“VLP-CspZ-WT”), or VLP-CspZ-Y207A/Y211A (“VLP-CspZ-Y207A/Y211A”) was mixed at indicated dilutions with guinea pig complement and 5 × 10^5^ cells/mL *Borrelia burgdorferi* strain B31-A3. The serum collected from three C3H/HeN mice at 42 days post inoculation of PBS was also included as negative control. Surviving spirochetes were quantified using dark-field microscopy after 24-h of incubation. **(A)** The survival percentage was derived from the proportion of serum-treated to untreated *B. burgdorferi*. Data shown are the mean ± SEM of survival percentage derived from three fields under the microscope for each sample. **(B)** The 50% borreliacidal titer of each serum sample, representing the dilution rate of the serum that effectively killed 50% of spirochetes, was obtained from the cure-fitting in Panel **(A)** (see Materials and Methods). Data shown are the mean ± SEM of borreliacidal titers of each serum sample derived from five CspZ, VLP-CspZ, or VLP-CspZ-Y207A/Y211A mice per group. The 50% borreliacidal titers of the serum samples from PBS- or VLP-inoculated mice were not detectable (“ND”) as those serum samples displayed no bactericidal activity. Statistical significances (*p* < 0.05) of differences in bactericidal titers relative to CspZ-immunized mice were determined using a *t*-test and are indicated (“*”).

Sera from mice immunized with VLP-CspZ-Y207A/Y211A eradicated spirochetes more effectively than that from CspZ- or VLP-CspZ-inoculated mice. Although antibody titers obtained with unmodified and modified CspZ were similar, the ability of these antibodies in killing spirochetes may be different. We thus examined if eliminating FH binding or VLP conjugation to CspZ would elicit more robust borreliacidal antibody responses. Stepwise dilutions of serum from PBS-, VLP-, CspZ-, VLP-CspZ-, or VLP-CspZ-Y207A/Y211A-inoculated mice were mixed with guinea pig complement and *B. burgdorferi*, and the levels of spirochete survival were quantified after 24-h incubation. The 50% borreliacidal activity (the dilution rate in which 50% of spirochetes are eliminated) was calculated to quantitatively compare the borreliacidal differences of these sera. Whereas the serum from the PBS- or VLP-inoculated mice was incapable of eradicating spirochetes, the serum from CspZ-, VLP-CspZ-, or VLP-CspZ-Y207A/Y211A-immunized mice killed *B. burgdorferi* in a dose-dependent manner (Figure 3A). The serum from CspZ-vaccinated mice killed 50% of spirochetes at an average dilution rate of 1:43, whereas diluting the serum from VLP-CspZ-immunized mice at an average of 1:143 eliminated 50% of *B. burgdorferi* (threefold more effective than that from CspZ-vaccinated mice; Figure 3A; Table S1 in Supplementary Material). Interestingly, the serum from the VLP-CspZ-Y207A/Y211A-immunized mice eradicated 50% of spirochetes at the average dilution rate of 1:395, which was ninefold or threefold more effective than that from the mice immunized with CspZ or VLP-CspZ, respectively (Figure 3; Table S1 in Supplementary Material). Our findings suggest that vaccination of VLP-CspZ-Y207A/Y211A induces antibodies with the greatest borreliacidal activity.

### Passive Immunization of Naïve Mice with Serum from VLP-CspZ-Y207A/Y211A-Vaccinated Mice Prevented Lyme Disease

We next determined if passively immunizing mice with serum containing anti-CspZ antibodies with greater borreliacidal activity provides more effective protection against Lyme infection. Naïve mice were passively immunized with serum collected from VLP, CspZ, VLP-CspZ, or VLP-CspZ-Y207A/Y211A actively immunized mice or the pre-immune mouse serum, and then infected with *B. burgdorferi* (Figure S1B in Supplementary Material). As expected, the pre-immune mouse serum did not protect mice against *B. burgdorferi* infection (0/6; Table 1). The serum from VLP-immunized mice was unable to protect any passively immunized mice from being infected by spirochetes (0/6; Table 1). Similarly, no protection was observed in any mice passively immunized with serum from CspZ-vaccinated mice (0/6; Table 1). Passive immunization with serum from VLP-CspZ-vaccinated mice prevented Lyme infections in 33% of mice (2/6), but this protection efficiency is not statistically different from that in pre-immune serum inoculated mice (*p* = 0.22; Table 1). Interestingly, passively immunizing with the serum obtained from VLP-CspZ-Y207A/Y211A-vaccinated mice protected 100% of mice from Lyme infection (6/6; Table 1), and such efficiency is significantly greater than that in pre-immune mouse serum-inoculated mice (*p* = 0.002; Table 1). These results suggest that the serum from the mice vaccinated with VLP-CspZ-Y207A/Y211A completely protects naïve mice from Lyme infection *via* passive immunization.

**Table 1:** Protection against *Borrelia burgdorferi* in mice passively immunized with serum raised from CspZ- or virus-like particle (VLP)-immunized mice.

Immunogen	No. of tissue culture positive/total^a^					No. of mice protected/total^a^^,^^b^	*p*-Value^c^
	Inoc. site	Bladder	Heart	Joint	Ear		
Preimmune serum	6/6	6/6	6/6	6/6	6/6	0/6	
VLP	6/6	6/6	6/6	6/6	5/6	0/6	1.00
CspZ	6/6	5/6	6/6	4/6	4/6	0/6	1.00
VLP-CspZ	4/6	4/6	4/6	4/6	4/6	2/6	0.22
VLP-CspZ-Y207A/Y211A	0/6	0/6	0/6	0/6	0/6	6/6	0.002

*^a^Combined two trials*.

*^b^Mice were considered infected (not protected) when at least one culture was positive*.

*^c^One-tailed Fisher Exact Probability Test, Compared to the mice inoculated with pre-immune mouse serum*.

### Immunization with VLP-CspZ-Y207A/Y211A Provided Greater Protection from Lyme-Associated Arthritis than Vaccination with CspZ or VLP-CspZ

**Figure 4:**
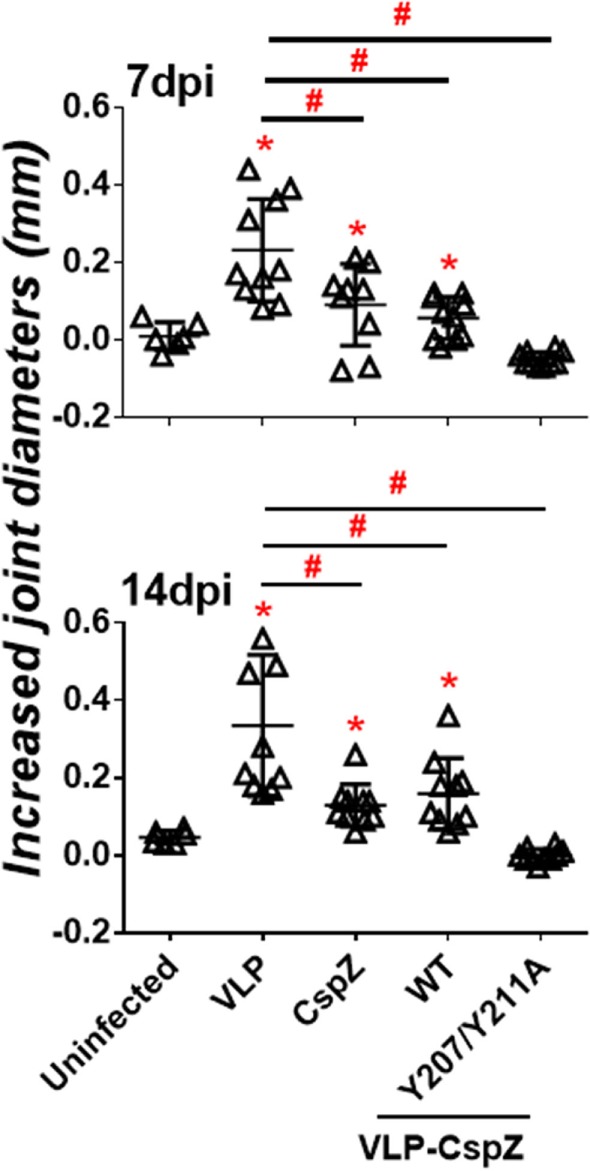
Immunizing mice with VLP-CspZ-Y207A/Y211A prevented joint swelling compared to virus-like particle (VLP) or VLP-CspZ vaccination. Ten C3H/HeN mice were vaccinated with VLP (“VLP”), CspZ (“CspZ”), VLP-CspZ (“VLP-CspZ-WT”), or VLP-CspZ-Y207A/Y211A (“VLP-CspZ-Y207A/Y211A”) prior to infection with 10^4^ *Borrelia burgdorferi* strain B31-A3. The diameters of tibiotarsus joints were measured at (*top panel*) 7 and (*bottom panel*) 14 days post-infection, and from uninfected mice of the same age. The joint size of six uninfected mice was also included as negative control. The increased joint diameters were derived from subtracting the group average tibiotarsus joint diameter prior to infection (0 days post-infection). Data shown are the mean ± SD of 6 (uninfected) or 10 (all others) mice per group. Statistical significance (*p* < 0.05) of differences in tibiotarsus joint diameters of each group relative to uninfected mice were determined using a one-way ANOVA test and *post hoc* analysis and are indicated (“*”). Significant differences (*p* < 0.05) between infected groups are indicated (“^#^”).

To test whether the CspZ antibodies with greater borreliacidal activity confer more efficient protection from Lyme arthritis *via* active immunization, C3H/HeN mice were actively immunized with VLP, CspZ, VLP-CspZ, or VLP-CspZ-Y207A/Y211A (Figure S1A in Supplementary Material). Joint diameters were measured at 7 and 14 days post infection (Figure S1C in Supplementary Material), as Lyme-induced joint swelling is detectable as early as these time points (33). C3H/HeN mice at the age group of 3- to 4-week old infected with 10^4^ of *B. burgdorferi* strain B31 have been previously shown to develop apparent swelling at tibiotarsus joint after 2 weeks of infection (36). Similarly, we observed that the VLP-inoculated mice at the similar age group and the identical infection dose of same spirochete strain also displayed tibiotarsus joint swelling, with the levels most apparent at 7 and 14 days post infection (at least eightfold greater joint diameters than uninfected mice; Figure 4). CspZ and VLP-CspZ vaccinations reduced joint swelling at these time points (approximately two fold less than the mice inoculated with VLP). However, the joint diameters were still significantly greater than that of uninfected mice (*p* < 0.05), suggesting that CspZ or VLP-CspZ vaccination was incapable of completely alleviating the joint swelling to the levels of uninfected mice (Figure 4). Interestingly, the joint diameters in the mice immunized with VLP-CspZ-Y207A/Y211A were at least threefold less than VLP-immunized mice at 7 and 14 days post infection, but were no different than uninfected mice (Figure 4). Our results imply that vaccination of VLP-CspZ-Y207A/Y211A reduces the joint swelling to the levels of uninfected mice during Lyme disease infection.

**Figure 5:**
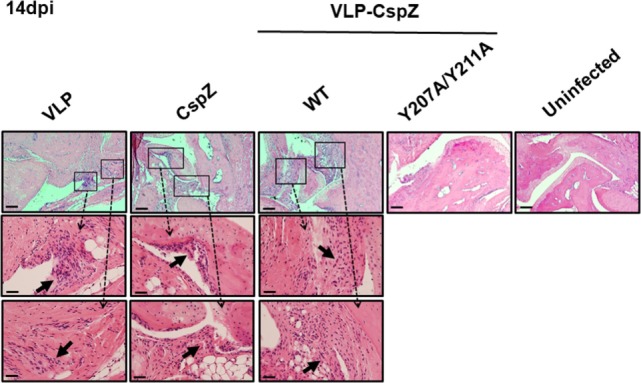
VLP-CspZ-Y207A/Y211A immunization prevents Lyme arthritis in *Borrelia burgdorferi-*infected C3H/HeN mice at levels similar to uninfected mice. Three C3H/HeN mice were vaccinated with virus-like particle (“VLP”), CspZ (“CspZ”), VLP-CspZ (“VLP-CspZ-WT”), or VLP-CspZ-Y207A/Y211A (“VLP-CspZ-Y207A”) and subsequently infected with 10^4^ *B. burgdorferi* strain B31-A3. Tibiotarsus joints were collected 14 days post infection and also from three uninfected mice of the same age. To assess inflammation, tissues were fixed and stained with hematoxylin and eosin. The representative images from one mouse per group are shown here. Top panels are lower-resolution images [joint, 10× (bar, 160 µm)]; bottom panels are higher-resolution images [joint, 2 × 20 (bar, 80 µm)] of selected areas (insets in top panels). Arrows indicate infiltration of immune cells.

Additionally, we histologically examined the severity of the arthritis in the mice vaccinated with VLP, CspZ, VLP-CspZ, or VLP-CspZ-Y207A/Y211A at 14 days post infection. VLP-inoculated mice developed apparent arthritis with inflammation at the joint, in which inflammatory cells infiltrated around the synovium (Figure 5). A similar arthritis phenotype was observed in CspZ- or VLP-CspZ-vaccinated mice (Figure 5). However, VLP-CspZ-Y207A/Y211A-vaccinated mice did not develop arthritis, with histopathology revealing inflammation similar to uninfected mice (Figure 5). C3H/HeN mice with the similar age group in this study infected with *B. burgdorferi* have been shown to display significant arthritis (36). Our finding thus suggests that vaccination of VLP-CspZ-Y207A/Y211 prevents mice from developing arthritis during Lyme infection.

### Immunization with VLP-CspZ-Y207A/Y211A Conferred Greater Protection against *B. burgdorferi* Tissue Colonization than CspZ or VLP-CspZ Vaccination

**Figure 6:**
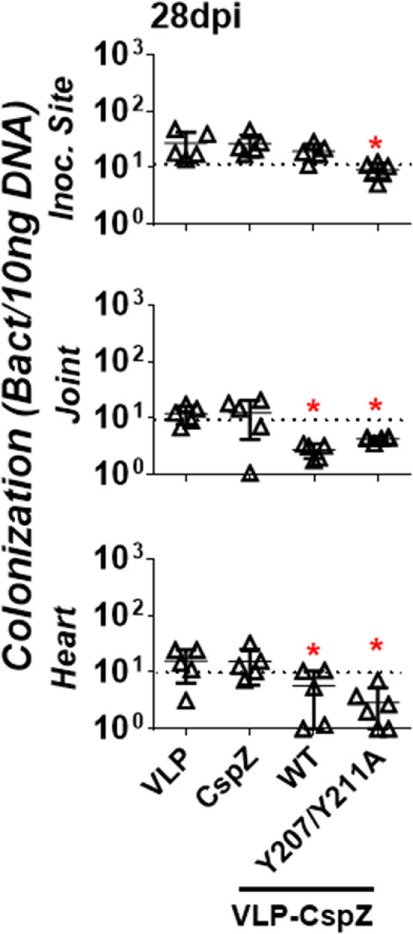
Vaccinating mice with VLP-CspZ-Y207A/Y211A eliminated *Borrelia burgdorferi* tissue colonization compared to virus-like particle (VLP) or VLP-CspZ immunization. C3H/HeN mice were immunized with VLP (“VLP”), CspZ (“CspZ”), or VLP-CspZ (“VLP-CspZ-WT”, five mice per group), or VLP-CspZ-Y207A/Y211A (“VLP-CspZ-Y207A/Y211A”, six mice per group) and subsequently infected with 10^4^ *B. burgdorferi* strain B31-A3. Spirochete colonization at inoculation site of skin (“inoc. site”, *top panel*), knee joint (“joint”, *middle panel*), and heart (“heart”, *bottom panel*) was quantitatively measured 28 days post infection. Colonization was derived by normalizing the number of spirochetes detected by quantitative PCR to 10 ng total DNA. Data shown are the mean ± SD, of five (VLP, CspZ, VLP-CspZ) or six (VLP-CspZ-Y207A/Y211A) mice. Statistical significance (*p* < 0.05) of differences in bacterial burden relative to VLP-immunized mice was determined using a one-way ANOVA test and *post hoc* analysis and are indicated (“*”).

To evaluate if vaccination with modified CspZ conjugated to VLP clears spirochete tissue colonization at later stages of infection, mice were actively immunized with VLP, CspZ, VLP-CspZ, or VLP-CspZ-Y207A/Y211A, prior to infection with *B. burgdorferi* (Figure S1A in Supplementary Material). Bacterial burdens were quantitatively assessed in tissues from these mice at 28 days post infection using qPCR (Figure S1C in Supplementary Material). *B. burgdorferi* strain B31 has been shown to colonize the inoculation site of skin, joints, and heart of C3H/HeN mice (at the levels approximately 10 to 100 spirochetes per 10 ng DNA) after infection by needles with 10^4^ of spirochetes for 28 days (30). When similar dose and the *B. burgdorferi* strain were introduced into the same age group of C3H/HeN mice, spirochetes also colonized these tissues of VLP-inoculated mice at similar levels (Figure 6; Table S2 in Supplementary Material; 12–27 spirochetes per 10 ng DNA). Consistent with previous findings (7, 11), we observed that *B. burgdorferi* colonized inoculation site of skin, joints, and heart of CspZ-immunized mice at a detectable level (Figure 6; Table S2 in Supplementary Material; 12–26 spirochetes per 10 ng DNA). This level was no different than that from VLP-inoculated mice (Figure 6). Further, the bacterial burdens in VLP-CspZ-immunized mice were below the detection limit in the heart and joints (detection limit = 10 bacteria copies per 10 ng DNA; Table S2 in Supplementary Material) and 2.7- to 4.4-fold lower than VLP-immunized mice (*p* < 0.05; Figure 6). However, there was no difference in the bacterial burden at the inoculation sites of VLP-CspZ and VLP-inoculated mice (Figure 6). Interestingly, vaccination of VLP-CspZ-Y207A/Y211A resulted in undetectable bacterial burdens at the inoculation site, joints, and heart during Lyme infection that were 2.8- to 5.4-fold lower than CspZ- and VLP-inoculated mice (*p* < 0.05; Figure 6). Our results indicate that immunization of VLP-CspZ-Y207A/Y211A reduces the spirochete colonization to an undetectable level during Lyme disease infection.

## Discussion

A number of strategies have been used to develop a Lyme disease vaccine, including inoculation of dead or live spirochetes (20), or recombinant proteins from *B. burgdorferi* or *Ixodes* ticks (37). In this study, we chose *B. burgdorferi* CspZ as a potential vaccine candidate because of its antigenicity and its ability to facilitate evasion of complement system (4, 7, 11). While vaccination with CspZ elicits a robust antibody response, it does not protect mice from Lyme infection, possibly due to insufficient functional antibodies (i.e., bactericidal) (7, 11). We thus re-evaluated the efficacy of CspZ as a vaccine against Lyme disease by conjugating CspZ to VLP to generate VLP-CspZ, and combined this approach with eliminating the FH-binding activity of CspZ to generate VLP-CspZ-Y207A/Y211A (6). Conjugating antigens to the highly repetitive structures of VLPs may alter the topology of these antigens. This may eventually allow B cells to more efficiently recognize the epitopes and develop greater levels of antibodies with enhanced bactericidal activity (38, 39). In fact, vaccinating mice with other *B. burgdorferi* outer surface proteins OspA or OspC conjugated to VLP induces robust levels of protective antibody response (40, 41). Consistent with these findings, though neither VLP-CspZ nor VLP-CspZ-Y207A/Y211A triggered greater titers of anti-CspZ antibodies compared to mice immunized with CspZ, immunizing mice with either of these VLP-CspZ proteins induced antibodies with robust levels of bacterial killing activity. Eliminating the ability of CspZ to bind FH exposes the FH-binding site and, therefore, may increase the ability of the epitopes close to/within this site to induce bactericidal antibodies. In fact, immunization with point mutants of a *Neisseria meningitidis* FH-binding protein fHbp with reduced FH-binding activity induces greater levels of bactericidal antibodies in vaccinated human FH-transgenic mice and in non-human primates than immunization with wild-type fHbp (19, 42–45). This reduction in immunogenicity as a result of binding to host proteins thus is not restricted to FH-binding molecules (46).

We then tested the combination of VLP conjugation and eliminated FH-binding activity of CspZ as a vaccine in protecting mice from Lyme disease infection *via* active and passive immunization. TiterMax Gold adjuvant has been used as this adjuvant was reported to induce greater and longer lasting titers than other adjuvants (29). Our data showed complete *in vivo* protection against Lyme disease from passive immunization with VLP-CspZ-Y207A/Y211A, but not CspZ or VLP-CspZ. During active immunization, CspZ-immunized mice partially alleviated joint swelling compared to the mice inoculated VLP after infection with *B. burgdorferi*. This finding appears to contradict with a previous study in which no difference in joint swelling was observed between unvaccinated- and CspZ-vaccinated mice (7). However, the differences in methodologies and experimental design prevent direct comparison between these studies. For example, differences can be due to the infectious dose [10^4^ in this study vs 10^5^ in Ref. (7)] and the type of adjuvant [TiterMax Gold in this study vs Complete Freund’s adjuvant in Ref. (7)]. In spite of such differences, both studies found that vaccination with unmodified CspZ is ineffective at either preventing joint swelling (7) or reducing the joint swelling to the level of uninfected mice (Figure 4). In addition, vaccination with CspZ or VLP-CspZ did not prevent arthritis, which implies that the bactericidal ability of the antibodies induced by either of these proteins were insufficient in alleviating Lyme associated arthritis. Vaccination of VLP-CspZ-Y207A/Y211A prevented both joint swelling and arthritis, possibly due to the robust borreliacidal activity of the induced antibodies.

We also observed that *B. burgdorfer*i colonizes colonization at both proximal (inoculation site) and distal mouse tissues (heart and joints) of unmodified CspZ-immunized mice, which is in agreement with previous observations (7, 11). Inoculating mice with either VLP-CspZ or VLP-CspZ-Y207A/Y211A decreased *B. burgdorferi* colonization to an undetectable level at distal tissues. However, VLP-CspZ-Y207A/Y211A vaccination cleared colonization at the inoculation site while VLP-CspZ immunization did not. One of the possibilities addressing this difference is that the clearance of *B. burgdorferi* in the inoculation site may require the antibodies with more robust bactericidal activity (e.g., the antibody induced by VLP-CspZ-Y207A/Y211A immunization) to penetrate the tight structure of the skin capillaries into this tissue (47). In this study, we have demonstrated that recombinant CspZ with both conjugating to VLP and eliminated its FH-binding activity is a protective antigen against Lyme disease infection in a murine model. Mice have been widely used as a model to test the efficacy of Lyme disease vaccine [e.g., Ref. (20, 37, 40, 41)]. Additionally, the observations from the previous generation of Lyme disease vaccine performed on mice reflect to the efficacy of this vaccine in humans (48–50). Thus, the findings in this study may provide useful information for the development of Lyme disease vaccine used in humans. Further, the specific strategy of VLP conjugation and eliminating binding to the target ligand may also be applied to antigens of other bacterial pathogens, potentially serving as a general model for vaccination development to ultimately improve human health.

## Ethics Statement

All mouse experiments were performed in strict accordance with all provisions of the Animal Welfare Act, the Guide for the Care and Use of Laboratory Animals, and the PHS Policy on Humane Care and Use of Laboratory Animals. The protocol (Docket Number 16-451) was approved by the Institutional Animal Care and Use Agency (IACUC) of Wadsworth Center, New York State Department of Health. All efforts were made to minimize animal suffering.

## Author Contributions

All authors contributed to the design and analysis of experiments. AM, IL, SK, XY, and Y-PL performed the experiments in this manuscript. AM, XY, PK, UP, Y-PL, and KT wrote the manuscript. All authors critically reviewed the manuscript.

## Conflict of Interest Statement

The authors declare that the research was conducted in the absence of any commercial or financial relationships that could be construed as a potential conflict of interest.

## Abbreviations

VLP, virus-like particle; VLP-CspZ, VLP-conjugated CspZ; VLP-CspZ-Y207A/Y211A, VLP-conjugated CspZ-Y207A/Y211A; *B. burgdorferi, Borrelia burgdorferi* sensu stricto; FH, Factor H; FHL-1, Factor H-like protein 1; CRASP-2, complement regulator-acquiring surface protein 2; A.U., arbitrary unit; qPCR, quantitative PCR; IACUC, Institutional Animal Care and Use Agency.
