# Supplementary figures and images for: Eliminating Factor H-Binding Activity of Borrelia burgdorferi CspZ Combined with Virus-Like Particle Conjugation Enhances Its Efficacy as a Lyme Disease Vaccine
Source: Front Immunol. 2018 Feb 8;9:181. doi: 10.3389/fimmu.2018.00181 (PMC5809437; doi:10.3389/fimmu.2018.00181)

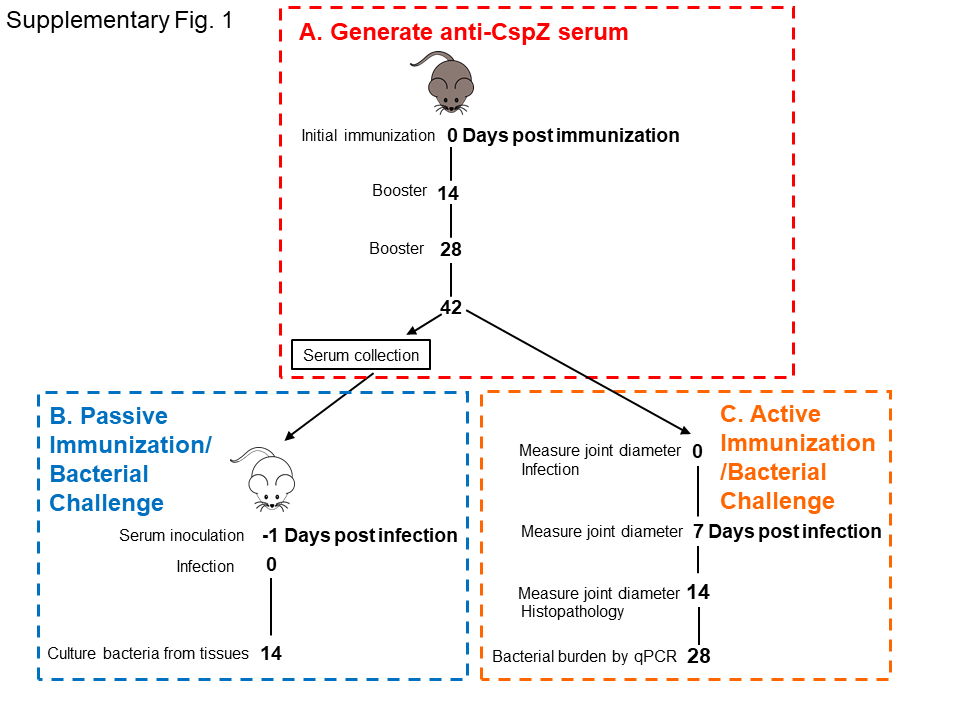

Supplement: Supplementary file 1 [file image_1.tif]

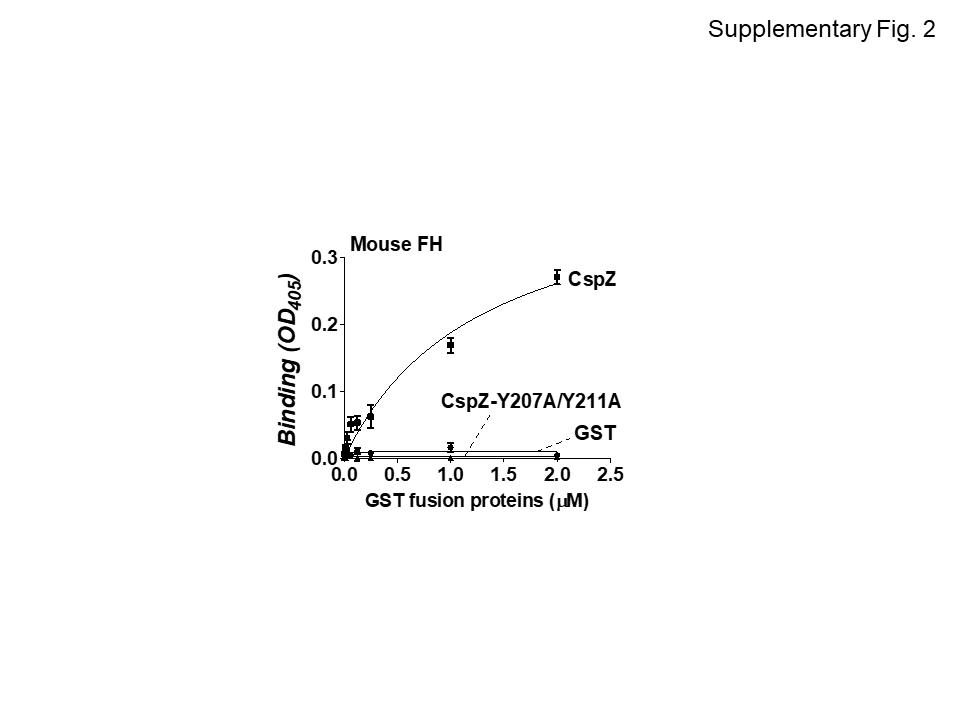

Supplement: Supplementary file 2 [file image_2.tif]
