# Supplementary material for: Eliminating Factor H-Binding Activity of Borrelia burgdorferi CspZ Combined with Virus-Like Particle Conjugation Enhances Its Efficacy as a Lyme Disease Vaccine
Source: Front Immunol. 2018 Feb 8;9:181. doi: 10.3389/fimmu.2018.00181 (PMC5809437; doi:10.3389/fimmu.2018.00181)
Supplement: Supplementary file 3 [file presentation_1.PDF]

Supplementary Material

Eliminating factor H-binding activity of *B. burgdorferi* CspZ combined with virus-like particle conjugation enhances its efficacy as a Lyme disease vaccine

Ashley Marcinkiewicz[§], Ilva Lieknina[§], Svetlana Kotelovica, Xiuli Yang, Peter Kraiczy, Utpal Pal, Yi-Pin Lin,* Kaspars Tars*

[§]These authors contributed equally to the work

*correspondence: Yi-Pin Lin, Ph.D.
Division of Infectious Disease,
Wadsworth Center, New York State Department of Health
120 New Scotland Ave, Albany, NY 12047
Telephone: 518-402-2233
Email: Yi-Pin.Lin@health.ny.gov

*correspondence: Kaspars Tars, Ph.D.
Latvian Biomedical Research and Study Centre
Ratsupites Str. 1 k-1, Riga, Latvia, LV-1067
Telephone: +371-678-08218
Email: Kaspars@biomed.lu.lv

Supplementary Figures and Tables

Supplementary Figures

Supplementary Figure 1. Experimental timeline of mouse immunization and infection. The timeline to **(A)** generate anti-CspZ serum, **(B)** passive immunization and bacteria challenge, and **(B)** active immunization and bacteria challenge was shown. **(A)** C3H/HeN mice received immunization and two boosters (14 and 28 days post immunization) of VLP, CspZ, VLP-CspZ, or VLP-CspZ-Y207A/Y211A, or PBS. Forty-two days after the initial immunization, sera from these mice were collected to detect their IgG or IgM titers and bactericidal activity. **(B)** Naive SW mice were inoculated with the serum from each group of the mice or pre-immune serum one day before bacteria challenge. These mice were then infected with 10^4 *B. burgdorferi* strain B31-A3. Tissues collected at 14 days post infection were placed in culture medium to determine the presence of *B. burgdorferi*. **(B)** C3H/HeN mice were received immunization and two boosters (14 and 28 days post immunization) of VLP, CspZ, VLP-CspZ, or VLP-CspZ-Y207A/Y211A, or PBS as shown in panel (A). Forty-two days after initial immunization, those mice were infected with 10^4 *B. burgdorferi* strain B31-A3. The diameter of the tibiotarsus joints were measured prior to infection as well as 7 and 14 days post infection. Mice were sacrificed 14 days post infection for histopathology, or 28 days post infection for bacterial burden quantification.

Supplementary Figure 2. Recombinant version of CspZ-Y207A/Y211A is incapable of binding to mouse FH. The indicated concentrations of GST tagged CspZ ("CspZ") or CspZ-Y207A/Y211A ("CspZ-Y207A/Y211A") or GST were added to triplicate wells coated with 1 µg of BSA (negative control, data not shown) or mouse FH, and protein binding was quantitated by ELISA (see Materials and Methods). The K_D value of CspZ to bind to mouse FH (0.72±0.42µM) was obtained by fitting the binding values to the equation described in Materials and Methods (R^2 = 0.9463). Numbers represent the mean ± standard deviation. Data represent the average of four replicates.

Supplementary Tables

Supplementary Table 1. Quantitative determination of borreliacidal activity in the serum obtained from CspZ-, VLP- or PBS-inoculated mice.

**Vaccination**	**50% borreliacidal titer**
**PBS**[a]	NI[c]
**VLP**[b]	NI
**CspZ**[b]	43.02±16.23
**VLP-CspZ**[b]	143.24±57.85
**VLP-CspZ-Y207A/Y211A**[b]	395.81±163.72

Data shown are mean ± standard error of the mean

[a] Three mice per group

[b] Five mice per group

[c] NI: No inhibition (no killing)

Supplementary Table 2. *B. burgdorferi* burden in tissues from CspZ-, VLP- or PBS-inoculated mice at 28 days post infection.

Immunogen	Colonization (Bacteria/10ng DNA)		
	**Inoculation Site**	**Joint**	**Heart**
**VLP**[a]	27.54 ± 15.27	12.01 ± 1.90	15.73 ± 4.15
**CspZ**[a]	26.92 ± 5.06	12.32 ± 3.63	15.62 ± 4.34
**VLP-CspZ**[a]	19.63 ± 2.93	2.72 ± 0.35*	5.77 ± 2.13*
**VLP-CspZ-Y207A/Y211A**[b]	9.30 ± 1.12*	4.29 ± 0.15*	2.94 ± 0.95*

Data shown are mean ± standard deviation of the number of *B. burgdorferi* present as determined from qPCR, based on the data in Figure 6.

[a] Five mice per group

[b] Six mice

* Bacterial burden are below the limit of detection (10 bacteria/10ng DNA), and significantly lower ($p < 0.05$) than VLP-vaccinated mice as determined with one-way ANOVA test and post-hoc analysis.
